# Antibodies to neurofascin, contactin-1, and contactin-associated protein 1 in CIDP: Clinical relevance of IgG isotype

**DOI:** 10.1212/NXI.0000000000000639

**Published:** 2019-11-21

**Authors:** Andrea Cortese, Raffaella Lombardi, Chiara Briani, Ilaria Callegari, Luana Benedetti, Fiore Manganelli, Marco Luigetti, Sergio Ferrari, Angelo M. Clerici, Girolama Alessandra Marfia, Andrea Rigamonti, Marinella Carpo, Raffaella Fazio, Massimo Corbo, Anna Mazzeo, Fabio Giannini, Giuseppe Cosentino, Elisabetta Zardini, Riccardo Currò, Matteo Gastaldi, Elisa Vegezzi, Enrico Alfonsi, Angela Berardinelli, Ludivine Kouton, Constance Manso, Claudia Giannotta, Pietro Doneddu, Patrizia Dacci, Laura Piccolo, Marta Ruiz, Alessandro Salvalaggio, Chiara De Michelis, Emanuele Spina, Antonietta Topa, Giulia Bisogni, Angela Romano, Sara Mariotto, Giorgia Mataluni, Federica Cerri, Claudia Stancanelli, Mario Sabatelli, Angelo Schenone, Enrico Marchioni, Giuseppe Lauria, Eduardo Nobile-Orazio, Jérôme Devaux, Diego Franciotta

**Affiliations:** From the Department of Brain and Behavioral Sciences (A.C., I.C., G.C., R.C., E.V.), University of Pavia, Pavia, Italy; Department of Neuromuscular Disease (A.C.), UCL Queen Square Institute of Neurology, London, United Kingdom; Neuroalgology Unit (R.L., P.D., L.P., G.L.), IRCCS Fondazione Istituto Neurologico “Carlo Besta,” Milan, Italy; Department of Neurosciences (C.B., M.R., A.S.), University of Padova, Padova, Italy; IRCCS Mondino Foundation (I.C., G.C., E.Z., R.C., M.G., E.V., E.A., A.B., D.F.), Pavia, Italy; Department of Neuroscience (L.B., C.D.M., A.S.), Rehabilitation, Ophthalmology, Genetics, Maternal and Child Health (DiNOGMI), University of Genova, Genova, Italy; IRCCS Ospedale Policlinico San Martino (L.B., C.D.M., A.S.), Genova, Italy; Department of Neurosciences (F.M., E.S., A.T.), Odontostomatological and Reproductive Sciences, University of Naples “Federico II,” Naples, Italy; Fondazione Policlinico Universitario Agostino Gemelli-IRCCS. UOC Neurologia (M.L., A.R., M.S.), Rome, Italy; Università Cattolica del Sacro Cuore (M.L., A.R., M.S.), Rome, Italy; Section of Neurology (S.F., S.M.), Department of Neuroscience, Biomedicine and Movement Sciences, University of Verona, Verona, Italy; Department of Neurology and Stroke Unit (A.M.C.), Ospedale di Circolo/Fondazione Macchi, Varese, Italy; Department of Systems Medicine (G.A.M., G.M.), University of Rome Tor Vergata, Rome, Italy; Neurological Department (A.R.), ASST Lecco; Ospedale Treviglio ASST Bergamo Ovest (M.C.), Italy; Department of Neurology (R.F., F.C.), San Raffaele Scientific Institute, Milan, Italy; Department of Neurorehabilitation Sciences (M.C.), Casa Cura Policlinico (CCP), Milan, Italy; Department of Clinical and Experimental Medicine (A.M.), University of Messina, Messina, Italy; Department of Medicine, Surgery and Neurosciences (F.G.), University of Siena, Italy; Referral Center for Neuromuscular Diseases and ALS (L.K., E.M.), AP-HM, Timone University Hospital, Marseille, France; Université de Bordeaux (C.M.), Interdisciplinary Institute for Neuroscience, Bordeaux, France; Humanitas Clinical and Research Center (C.G., P.D., E.N.-O.), Milan University, Milan, Italy; IRCCS Centro Neurolesi “Bonino Pulejo” (C.S.), Messina, Italy; Department of Biomedical and Clinical Sciences “Luigi Sacco” (G.B., G.L.), University of Milan, Milan, Italy; and Institute for Neurosciences of Montpellier (J.D.), INSERM U1051, Montpellier University, Hopital Saint Eloi, Montpellier, France.

## Abstract

**Objective:**

To assess the prevalence and isotypes of anti-nodal/paranodal antibodies to nodal/paranodal proteins in a large chronic inflammatory demyelinating polyradiculoneuropathy (CIDP) cohort, compare clinical features in seronegative vs seropositive patients, and gather evidence of their isotype-specific pathogenic role.

**Methods:**

Antibodies to neurofascin-155 (Nfasc155), neurofascin-140/186 (Nfasc140/186), contactin-1 (CNTN1), and contactin-associated protein 1 (Caspr1) were detected with ELISA and/or cell-based assay. Antibody pathogenicity was tested by immunohistochemistry on skin biopsy, intraneural injection, and cell aggregation assay.

**Results:**

Of 342 patients with CIDP, 19 (5.5%) had antibodies against Nfasc155 (n = 9), Nfasc140/186 and Nfasc155 (n = 1), CNTN1 (n = 3), and Caspr1 (n = 6). Antibodies were absent from healthy and disease controls, including neuropathies of different causes, and were mostly detected in patients with European Federation of Neurological Societies/Peripheral Nerve Society (EFNS/PNS) definite CIDP (n = 18). Predominant antibody isotypes were immunoglobulin G (IgG)4 (n = 13), IgG3 (n = 2), IgG1 (n = 2), or undetectable (n = 2). IgG4 antibody-associated phenotypes included onset before 30 years, severe neuropathy, subacute onset, tremor, sensory ataxia, and poor response to intravenous immunoglobulin (IVIG). Immunosuppressive treatments, including rituximab, cyclophosphamide, and methotrexate, proved effective if started early in IVIG-resistant IgG4-seropositive cases. Five patients with an IgG1, IgG3, or undetectable isotype showed clinical features indistinguishable from seronegative patients, including good response to IVIG. IgG4 autoantibodies were associated with morphological changes at paranodes in patients' skin biopsies. We also provided preliminary evidence from a single patient about the pathogenicity of anti-Caspr1 IgG4, showing their ability to penetrate paranodal regions and disrupt the integrity of the Nfasc155/CNTN1/Caspr1 complex.

**Conclusions:**

Our findings confirm previous data on the tight clinico-serological correlation between antibodies to nodal/paranodal proteins and CIDP. Despite the low prevalence, testing for their presence and isotype could ultimately be part of the diagnostic workup in suspected inflammatory demyelinating neuropathy to improve diagnostic accuracy and guide treatment.

**Classification of evidence:**

This study provides Class III evidence that antibodies to nodal/paranodal proteins identify patients with CIDP (sensitivity 6%, specificity 100%).

Chronic inflammatory demyelinating polyradiculoneuropathy (CIDP), the most commonly acquired inflammatory neuropathy worldwide, is clinically heterogeneous. Proven treatments for CIDP include corticosteroids, plasma exchange, and intravenous immunoglobulin (IVIG). The response rates to treatments were reported to be heterogeneous in subgroups of patients, and the availability of specific biomarkers could provide guidance for patient-tailored immunotherapeutic options.^1^

Antibodies to cell adhesion molecules of the paranodal complex, neurofascin-155 (Nfasc155), contactin-1 (CNTN1), and contactin-associated protein 1 (Caspr1), and to nodal neurofascin-140/186 (Nfasc140/186) have been identified in various percentages of patients with CIDP, with IgG4 being the predominant isotype of these antibodies.^2–8^ Moreover, IgG4-seropositive patients show specific clinical features and a poor response to IVIG.

The prevalence of anti-CNTN1 and Nfasc155 IgG4 has been well documented in cohorts of Japanese patients.^2,5,6,9,10^ It has been shown that anti-CNTN1 IgG4 antibodies are pathogenic in animal models and have a function-blocking activity.^11,12^ Moreover, anti-CNTN1 and Nfasc155 IgG4 are associated with specific alterations of the paranodal axo-glial contacts in nerve biopsies,^13,14^ suggesting that these antibodies induce conduction defects in patients by altering paranode integrity, hence the term paranodopathy. In Europe, the study of these antibodies has been so far limited to small cohorts of Spanish, French, and German patients with CIDP,^3,4,15,16^ and there is little information on the role and associated clinical features of antibodies of the IgG1-3 isotype. Moreover, the prevalence of anti-Caspr1 antibodies is still largely unknown, as well as their possible pathogenic role.

Here, we assessed the prevalence and isotypes of antibodies against Nfasc155, Nfasc140/186, CNTN1, and Caspr1 in a large cohort of Italian patients affected by CIDP to better characterize their clinical associations. We also investigated skin biopsies from antibody-reactive patients for morphological abnormalities and the pathogenicity of anti-Caspr1 antibodies using in vitro and in vivo models.

## Methods

### Patients and sera

The primary research question of this study is to test the frequency of antibodies to nodal/paranodal proteins in CIDP (Class III evidence). Sera from 342 patients fulfilling the diagnostic criteria for CIDP^17^ (306 typical and 36 atypical) were collected in 11 Italian centers with specific expertise in neuromuscular disorders and were all tested by ELISA and cell-based assay (CBA) for antibodies to Nfasc155 and CNTN1 and by CBA only for antibodies to Nfasc140/186 and Caspr1 by 2 independent laboratories (Istituto di Ricovero e Cura a Carattere Scientifico [IRCCS] Mondino Foundation, National Neurological Institute of Pavia, Italy, and Institute for Neurosciences of Montpellier, France). As controls, samples from healthy controls (HCs, n = 60); patients with Guillain-Barré syndrome (GBS, n = 31), multifocal motor neuropathy (MMN, n = 13), inherited neuropathy (n = 18), other noninflammatory neuropathy (n = 52) including paraproteinemic neuropathy (n = 18) and toxic-metabolic neuropathy (n = 34), and patients with MS (n = 60) were tested. Methods for ELISA^18^ and CBA^6,10^ were previously reported and are available as supplementary methods, links.lww.com/NXI/A165. Clinical information was collected from seropositive patients with CIDP and from 64 randomly selected seronegative patients with CIDP. Weakness was graded as mild if the medical research council (MRC) score of the weakest muscle considered of either proximal or distal muscle groups was <5 and >3, moderate if MRC = 3, or severe if MRC <3. Disability was graded using the Overall Neuropathy Limitation Scale (ONLS).^19^ Response to treatment was investigated post hoc and was defined as a persistent improvement of disability with an ONLS change after treatment ≥1. Reactivity of sera to teased fibers from murine sciatic nerves was also evaluated as previously described.^2^

### Immunohistochemistry on skin biopsy

Three patients with anti-Nfasc155 IgG4 antibodies, 1 patient with anti-CNTN1 IgG3/IgG4 antibodies, 1 patient with anti-Caspr1 IgG4 antibodies, 1 patient with anti-Nfasc155 antibodies of an undetectable isotype, and 6 seronegative patients with CIDP underwent 3-mm punch skin biopsies at 1 distal site of the lower limb as per EFNS/PNS guidelines.^20^ Nodal/internodal morphological analysis was performed by double immunofluorescence staining for myelin basic protein and Nfasc or myelin basic protein and Caspr1. Detailed methods are available in supplementary methods, links.lww.com/NXI/A165.

### Purification of patients' antibodies

For in vitro and in vivo studies, IgG1 and IgG4 were purified from the serum of 1 Caspr1-reactive patient and the plasma of a HC and their pathogenicity was tested by intraneural injection on murine sciatic nerve and cell aggregation assay. Methods for in vitro and in vivo studies are detailed in supplementary methods, links.lww.com/NXI/A164.

### Statistics

Clinical data were presented as number (%) or mean (min-max). Categorical variables were compared in seronegative and seropositive patients using the χ^2^ test or Fisher exact test; unpaired 2-tailed Student *t* test was used for comparing continuous variables. Analysis of clinical variables was performed using STATA. *p* values inferior to 0.05 were considered significant.

### Standard protocol approvals, registrations, and patient consents

The study was approved by local institutional ethical committees. Written informed consent was obtained from all patients (or guardians of patients) participating in the study.

### Data availability

Anonymized data from this study will be shared by request from any qualified investigator.

## Results

### Serologic findings

**Figure 1:**
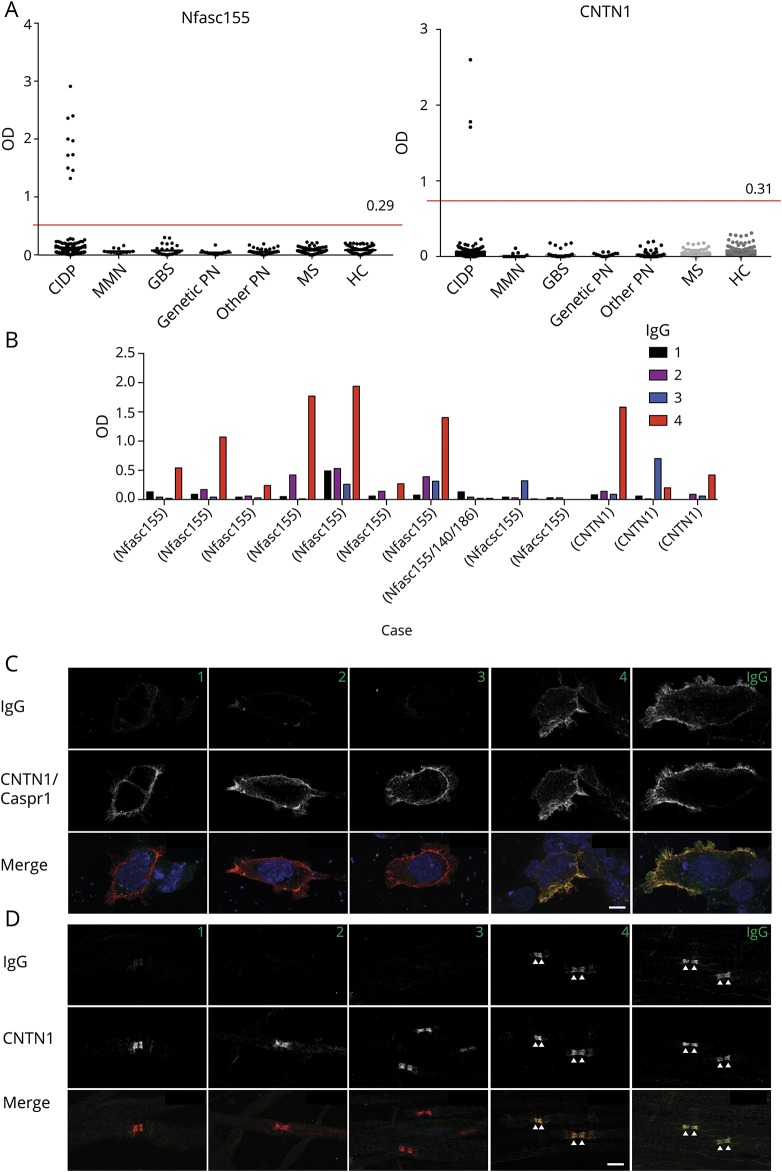
Reactivity to Nfasc155, CNTN1, and Caspr1 in CIDP by ELISA and CBA (A) Serum samples from patients with CIDP (n = 342), MMN (n = 13), GBS (n = 31), genetic PN (n = 18), other noninflammatory PN (n = 52), MS (n = 60) and from HCs (n = 60) were tested for autoantibodies to human Nfasc155 (left) and CNTN1 (right) by ELISA. OD are shown after subtraction of the baseline OD reading to bovine serum albumin. The red line represents the mean OD in HCs plus 3 standard deviations. (B) IgG isotype in Nfasc155- and CNTN1-positive patients. (C) The sera (here case 14) were tested on living HEK cells transfected with CNTN1 and Caspr1 (red) and then revealed with mouse antihuman IgG1, IgG2, IgG3, or IgG4 (green) as indicated. Nuclei were stained with DAPI (blue). (D) These are teased fibers from mouse sciatic nerves immunostained for CNTN1 (red) and the serum from case 14, then revealed with mouse antihuman IgG1, IgG2, IgG3, or IgG4 (green) as indicated. IgG1 and IgG4 from this patient reacted against Caspr1 and bound to the paranodal regions. Scale bars: 10 μm. Caspr1 = contactin-associated protein 1; CBA = cell-based assay; CIDP = chronic inflammatory demyelinating polyradiculoneuropathy; CNTN1 = contactin-1; GBS = Guillain-Barré syndrome; HC = healthy control; HEK = human embryonic kidney; MMN = multifocal motor neuropathy; Nfasc155 = neurofascin-155; OD = Optical density; PN = peripheral neuropathy.

Of 342 sera from patients with CIDP tested, 10 (3%) were positive for antibodies to anti-Nfasc155 using ELISA and CBA (figure 1A). IgG isotypes were IgG4 in 7, IgG3 in 1, and undetectable in 2 patients (figure 1B). One of these latter patients showed IgG4 reactivity against Nfasc140/186 and was thus considered a Nfasc140/186-reactive patient. Three patients (1%) tested positive on ELISA and CBA for anti-CNTN1 antibodies (figure 1A). IgG isotypes were IgG4 in 2 patients and mixed IgG3/IgG4 in 1 patient (figure 1B). All sera reactive to Nfasc155 or CNTN1 by ELISA were also confirmed by CBA, and no nonspecific reactivities by ELISA only were identified. Six patients (2%) showed reactivity against Caspr1 on CBA (figure 1C). Three had an IgG4-predominant isotype, 2 had an IgG1 isotype, and 1 did not have a detectable isotype. Overall, 19 patients (6%) had antibodies against one of these 4 targets. None of the HCs and patients with GBS, MMN, hereditary neuropathy, other noninflammatory neuropathies, or MS tested positive, indicating that the presence of antibodies to nodal/paranodal proteins accurately identify patients with CIDP with sensitivity 6% and specificity 100%. Of note, none of the sera from 36 atypical CIDP cases tested positive.

Sera from patients with IgG4 anti-Nfasc155, anti-CNTN1, or anti-Caspr1 antibodies, but not anti-Nfasc155 of the IgG3 isotype or undetectable isotype, consistently showed reactivity against paranodes when tested on teased fibers from murine sciatic nerves (figure 1D). Sera with anti-Nfasc140/186 IgG4 that co-reacted against Nfasc155 stained the nodes of Ranvier on teased fibers.

### Clinical features

#### Patients with anti-Nfasc155 IgG4 antibodies

Of patients with anti-Nfasc155 IgG4 antibodies, 4 were men and 3 were women, with an average age at onset of 31 ± 18 years (table e-1, links.lww.com/NXI/A164). The onset of neuropathy was subacute in 5 patients. One case was initially diagnosed with GBS.

All patients had symmetric 4-limb muscle weakness, which was mild to moderate in the upper limbs and proximal muscles of the lower limbs but severe in 5 patients in the lower limb distal muscles. Pinprick and position sense at the hallux were reduced or abolished in all patients. Tremor was also invariably present, and 6 had sensory ataxia. One patient reported neuropathic pain at onset, and 1 had dysphagia. One of them presented optic neuritis during steroid tapering. Brain MRI was thus performed but did not show evidence of disseminated inflammatory CNS disease. The neuropathy was moderately to severely disabling with an ONLS at onset of 5.8 ± 2.2. Two patients required a wheelchair 1 month after the disease onset. The patients received IVIG (n = 6), steroids (n = 7), azathioprine (n = 1), or rituximab (n = 1). Only 1 patient responded to IVIG and steroids (ONLS 4 → 2), 1 patient showed response to steroids (ONLS 6 → 2), and 1 patient unresponsive to IVIG and steroids had sustained response after rituximab (ONLS 9 → 2).

#### Patients with anti-CNTN1 IgG4 antibodies

Of patients with anti-CNTN1 IgG4 antibodies, 3 were men with a mean age at onset of 56 ± 26 years (table e-1, links.lww.com/NXI/A164). The onset of neuropathy was subacute in 2 patients. Strength was impaired in both proximal and distal muscles in the upper and lower limbs, often to a severe degree, and 2 of them required a wheelchair. Pinprick sensation and proprioception were either severely reduced or abolished, and all of them had sensory ataxia. One patient showed cranial nerve involvement and respiratory failure. One patient with anti-CNTN1 IgG3/IgG4 antibodies had a concurrent onset of CIDP and nephrotic syndrome because of membranous glomerulonephritis. A kidney biopsy showed subepithelial deposits of immune complexes and complement deposition. CNTN1 patients had the highest disability at onset with an ONLS of 7.6 ± 2.5. One patient died of cardiac disease 6 months after the disease onset. The patients were treated with IVIG (n = 3), steroids (n = 3), plasma exchange (n = 2), and immunosuppressive drugs (n = 2). None of them had a sustained response to either IVIG or steroids. Cyclophosphamide was effective in 2 patients, leading to significant improvement of the neuropathy in 1 case and complete remission of the neuropathy together with regression of the nephrotic syndrome in the other case, who did not require further treatment.

#### Patients with anti-Caspr1 IgG4 antibodies

Of seropositive patients with anti-Caspr1 IgG4 antibodies, 2 were men and 1 was a woman, with a mean age at onset of 43 ± 17 years (table e-1, links.lww.com/NXI/A164). The onset was subacute in 2 patients. Weakness was mild to moderate in proximal muscle groups and moderate to severe in distal muscle groups in both upper and lower limbs. Pinprick sensation was reduced, and position sense was abolished at the hallux in all of them. None of them reported neuropathic pain. One patient complained of dysphagia, and 1 patient had tremor. The disease was severe, with a mean ONLS of 8.3 ± 0.6. The patients were treated with IVIG (n = 3), steroids (n = 3), plasma exchange (n = 1), methotrexate (n = 1), and interferon-alfa (n = 1). A sustained response was observed only in 1 patient receiving steroids and methotrexate (ONLS 9 → 6) and 1 patient treated with interferon-alfa (ONLS 8 → 7).

#### Seropositive patients with an IgG1-3 isotype or undetectable IgG isotype

Six patients had IgG1 or IgG3 antibodies or an undetectable IgG isotype against Nfasc155 (n = 3) or Caspr1 (n = 3) (table e-1, links.lww.com/NXI/A164). One of these patients presented antibodies reacting to both Nfasc155 and Nfasc140/186. The clinical features of this patient, mainly consisting in a very early onset IVIG-responsive neuropathy, have been previously detailed.^16^ We will thus focus here on the 5 other patients, 2 men and 3 women. In 2 of them, both reactive against Caspr1, the disease started in the first decade of life. The mode of onset was subacute in 1 patient only. If present, weakness was graded as mild to moderate in upper limbs and proximal muscles of lower limbs. All 5 patients had weakness in lower limb distal muscles, which was severe in 2 of them. Pinprick sensation was reduced in 3 and position sense was reduced at the hallux in 2 patients, but none had absent proprioception. Sensory ataxia was reported in 4 patients, 2 with anti-Nfasc155 antibodies and 2 with anti-Caspr1 antibodies, whereas tremor was observed in 2 patients with anti-Caspr1 antibodies. Disability was usually low with a mean ONLS at onset of 3.2 ± 1.3. Only 1 case with anti-Caspr1 antibodies needed walking aids. The patients were treated with IVIG (n = 4) and steroids (n = 2). One patient with minimal disability did not require treatment. Three of 4 patients treated with IVIG had a good response.

#### Comparison of seropositive vs seronegative patients

We compared the clinical features and responses to treatments of patients with IgG4 antibodies against Nfasc155, CNTN1, or Caspr1 with those of 64 randomly selected patients with CIDP who were negative for antibodies against Nfasc155, Nfasc140/186, CNTN1, or Caspr1. We also compared seropositive patients with an IgG1, IgG3, or undetectable IgG isotype with seronegative patients (table 1).

**Table 1:**
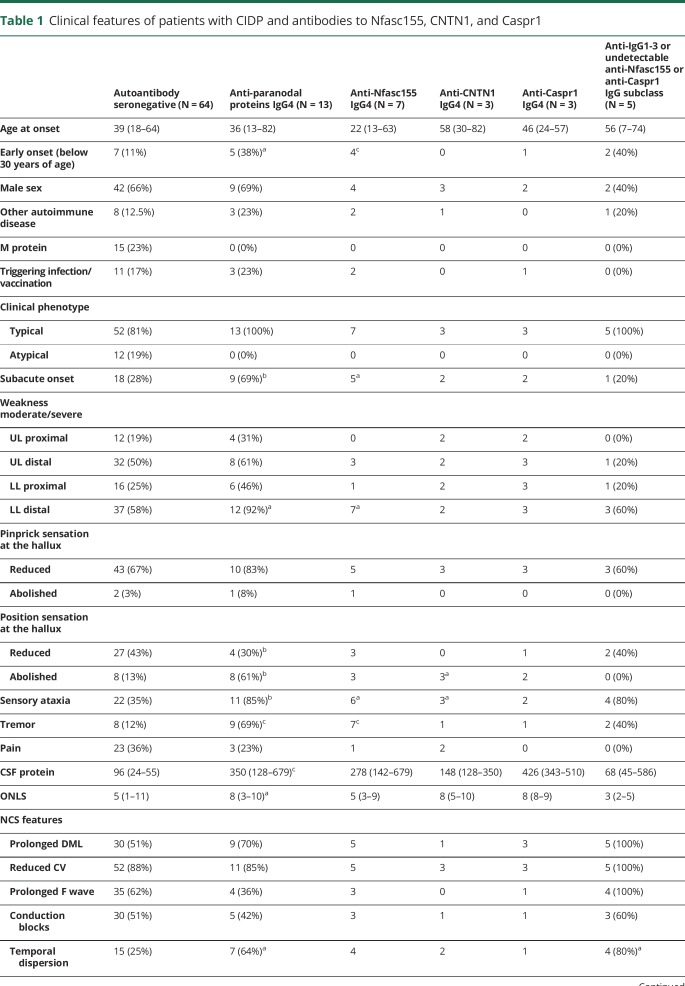

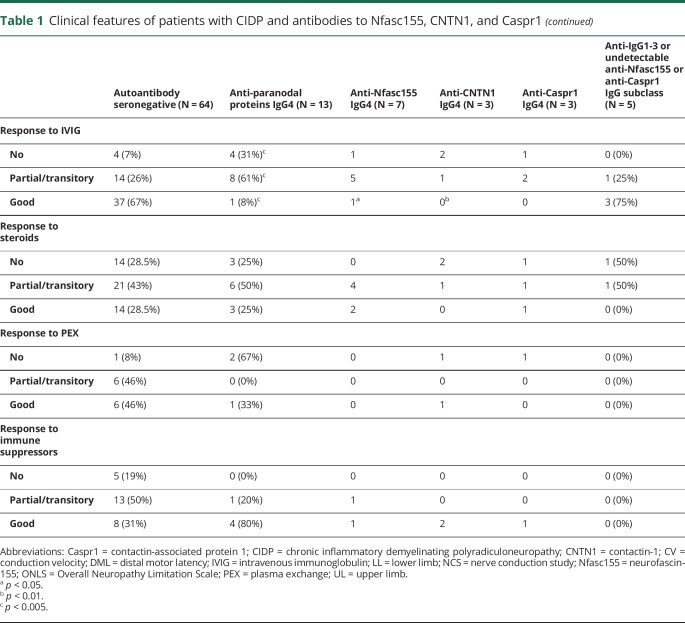
Clinical features of patients with CIDP and antibodies to Nfasc155, CNTN1, and Caspr1

Anti-Nfasc155 and anti-Caspr1 IgG4 seropositive patients had more frequently an early disease onset, before 30 years of age, as opposed to that of anti-CNTN1 IgG4 seropositive patients and seronegative patients. A subacute onset was reported in up to two-thirds of anti-Nfasc155, anti-CNTN1, and anti-Caspr1 IgG4 seropositive patients compared with 28% of seronegative patients. According to the EFNS/PNS criteria, CIDP phenotype was typical in all IgG4-seropositive patients. Tremor was invariably present in patients with anti-Nfasc155 IgG4 antibodies but was also observed in anti-CNTN1 and anti-Caspr1 IgG4 seropositive patients. Severe proprioceptive loss and sensory ataxia were significantly more frequent in IgG4-seropositive patients and were observed in all patients with anti-CNTN1 antibodies, 2 patients with anti-Caspr1 IgG4 antibodies, and 3 patients with anti-Nfasc155 IgG4 antibodies (table 1). In anti-CNTN1 and anti-Caspr1 IgG4 seropositive patients, weakness was usually graded as moderate to severe across proximal and distal muscle groups, whereas in patients with anti-Nfasc155 IgG4 antibodies, motor impairment showed a characteristic distal predominance in the lower limbs.

A higher level of CSF total protein and the presence of temporal dispersion on nerve conduction studies were also characteristic features of patients with autoantibodies of the IgG4 isotype. IgG4-seropositive patients had a higher disability at onset: 5 patients needed a walking aid, and 5 patients required a wheelchair. In particular, patients with anti-CNTN1 or anti-Caspr1 IgG4 antibodies showed the highest disability at onset.

Patients with anti-Nfasc155, CNTN1, or Caspr1 IgG4 antibodies had a lower response rate to IVIG compared with seronegative patients (8% vs 67%, *p* < 0.001), but there was no remarkable difference in the response to steroids or immunosuppressive treatment. In particular, 1 patient with anti-Nfasc155 IgG4 antibodies was treated with rituximab, 1 patient with anti-Nfasc155 IgG4 antibodies was treated with azathioprine, 2 patients with anti-CNTN1 IgG4 antibodies were treated with cyclophosphamide, and 1 patient with anti-Caspr1 IgG4 antibodies was treated with methotrexate. Four of them who were started on immunosuppressors within 1 year from disease onset showed long-lasting good response.

Patients with antibodies to Nfasc155, CNTN1, or Caspr1 of the IgG1, IgG3, or undetectable isotype did not show distinct clinical features or response to treatments compared with seronegative patients, except for more frequent temporal dispersion on nerve conduction studies.

#### Morphological changes of the nodes of Ranvier in skin biopsy of seropositive patients

**Figure 2:**
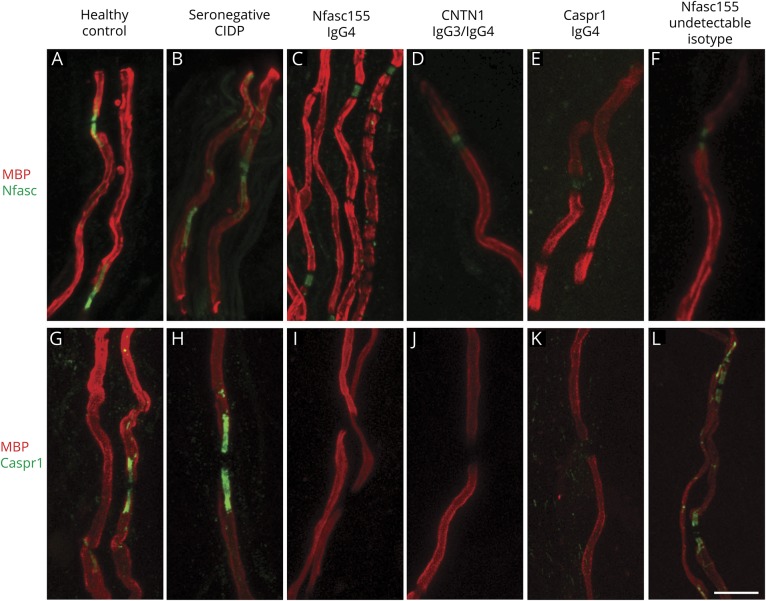
Morphological alterations of the nodes of Ranvier in patients with CIDP with IgG4 autoantibodies We evaluated skin biopsies from 3 patients with IgG4 anti-Nfasc155 antibodies, 1 patient with IgG3/IgG4 anti-CNTN1 antibodies, 1 patient with IgG4 anti-Caspr1 antibodies, 1 patient with undetectable isotype IgG anti-Nfasc155, and 6 seronegative patients with CIDP. Analysis of myelinated fibers showed elongation of the nodes of Ranvier and loss of paranodal Nfasc155 staining in skin biopsies from patients with anti-Nfasc155 (C) and Caspr1 (E) IgG4. Moderate elongation of the nodes of Ranvier and loss of Nfasc155 paranodal staining were also observed in myelinated fibers of a CNTN1 IgG3/IgG4-positive patient (D). Contrarily, we did not observe similar changes in the patient with undetectable isotype IgG anti-Nfasc155 antibodies (F), in seronegative patients with CIDP (B), or in HCs (A). A complete loss of Caspr1 staining was observed in biopsies from patients with IgG4 antibodies to paranodal proteins (I, L, M), but not in an Nfasc155 seropositive patient with an undetectable isotype (N) and in seronegative CIDP (H) or healthy patients (G). Caspr1 = contactin-associated protein 1; CIDP = chronic inflammatory demyelinating polyradiculoneuropathy; CNTN1 = contactin-1; HC = healthy control; Nfasc155 = neurofascin-155.

We evaluated skin biopsies from 3 patients with anti-Nfasc155 IgG4 antibodies, 1 patient with anti-CNTN1 IgG3/IgG4 antibodies, 1 patient with anti-Caspr1 IgG4 antibodies, 1 patient with anti-Nfasc155 antibodies of an undetectable isotype, and 6 seronegative patients with CIDP. Analysis of myelinated fibers from patients with anti-Nfasc155 and anti-Caspr1 IgG4 showed elongation of the nodes of Ranvier and loss of paranodal Nfasc155 and Caspr1 staining. Moderate elongation of the nodes of Ranvier and loss of Nfasc155 paranodal staining were also observed in myelinated fibers of the patient with anti-CNTN1 IgG3/IgG4 antibodies. Contrarily, we did not observe similar changes in the patient with anti-Nfasc155 antibodies with an undetectable isotype, seronegative patients CIDP, or HCs (figure 2).

#### Pathogenic effects of anti-Caspr1 IgG4 and IgG1 antibodies

**Figure 3:**
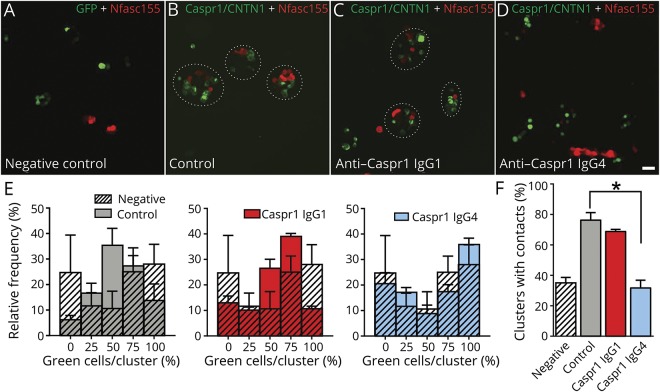
IgG4 to Caspr1 disrupt the interaction between Nfasc155 and CNTN1/Caspr1 (A–D) HEK cells transfected with CNTN1 and Caspr1 (green) or Nfasc155 (red) were incubated together for 2 hours in the presence of control IgG4 (B), anti-Caspr1 IgG1 (C), or anti-Caspr1 IgG4 (D). As negative controls, HEK cells transfected with Nfasc155 (red) were incubated with cells transfected with GFP (A). Anti-Caspr1 IgG4, but not anti-Caspr1 IgG1, abrogated the aggregation of Nfasc155-transfected cells with CNTN1/Caspr1. Scale bar: 10 μm. (E–F) The graphs represent the relative frequency of green cells per aggregates (n = 4 experiments for each condition). The percentage of cell clusters with contacts between green and red cells was quantified (F). The percentage of contacts was significantly decreased in the presence of anti-Caspr1 IgG4 (*p* < 0.005 by unpaired 2-tailed Student *t* tests and by one-way ANOVA, followed by Bonferroni post hoc tests). Bars represent mean and SEM. ANOVA = analysis of variance; Caspr1 = contactin-associated protein 1; CNTN1 = contactin-1; GFP = green fluorescent protein; HEK = human embryonic kidney; Nfasc155 = neurofascin-155.

Anti-CNTN1 IgG4 antibodies were previously found to have function-blocking activity and to disrupt the interaction between CNTN1 and its glial partner Nfasc155.^12^ We have previously shown that anti-CNTN1 IgG4 and anti-Nfasc155 IgG4 antibodies are pathogenic and disrupt the paranodal regions.^11,21^ We thus examined whether anti-Caspr1 antibodies also affect the integrity of the CNTN1/Caspr1/Nfasc155 complex. In particular, we purified IgG1 and IgG4 and tested the effects of the different antibody isotypes. First, we tested the impact of IgG4 and IgG1 fractions from Caspr1-positive patients on the interaction of CNTN1/Caspr1 with Nfasc155 by a cell aggregation assay (figure 3). Human embryonic kidney cells were transfected with Nfasc155 and mCherry and incubated for 2 hours with cells coexpressing CNTN1/Caspr1 and green fluorescent protein (GFP). The percentage of cell clusters showing contacts between red and green cells and the percentage of green and red cells per cell clusters were quantified. As a negative control, Nfasc155-mCherry-transfected cells were incubated with cells expressing GFP only. In the absence of CNTN1/Caspr1, Nfasc155-expressing cells do not form significant contact with GFP-expressing cells, and most clusters express only green or red cells. The expression of CNTN1/Caspr1 significantly increases the number of clusters with contacts, and most cell clusters show an average of 50% of CNTN1/Caspr1- and 50% of Nfasc155-expressing cells. The presence of 10 μg of IgG4 from a Caspr1-positive patient, but not IgG1, abolished the interaction between CNTN1/Caspr1- and Nfasc155-expressing cells to the level of negative controls, thus supporting a blocking function of anti-Caspr1 IgG4 antibodies.

**Figure 4:**
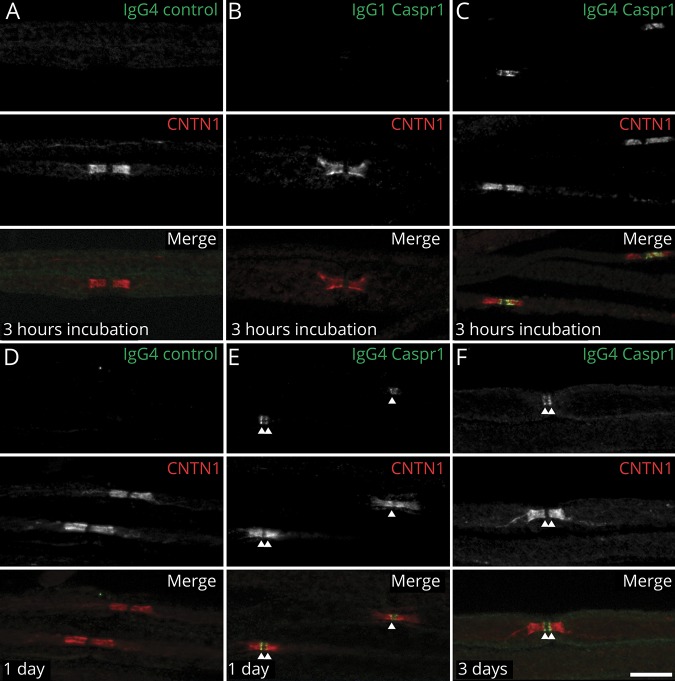
Anti-Caspr1 IgG4, but not IgG1, invades the paranodal regions (A–C) Sciatic nerve fibers were incubated in vitro with purified control IgG4 (A), anti-Caspr1 IgG1 (B), or anti-Caspr1 IgG4 (C) for 3 hours and immunolabeled for IgG (green) and CNTN1 (red). (D–F) Sciatic nerves were fixed 1 or 3 days after intraneural injections of purified control IgG4 (D), anti-Caspr1 IgG4 (E–F), immunolabeled for CNTN1 (red), and human IgG (green). Note that only anti-Caspr1 IgG4 penetrated the paranodes. One or 3 days after injection, IgG4 antibodies were detected at the paranode borders (arrows). Images are representative of 3 independent experiments. Scale bar: 10 μm. Caspr1 = contactin-associated protein 1; CNTN1 = contactin-1.

To further confirm these findings, mouse sciatic nerve segments were incubated in vitro with 10 μg of IgG4 or IgG1 fractions from Caspr1-positive patients for 3 hours, and IgG deposition was monitored. IgG4 but not IgG1 antibodies were found to penetrate the paranodal regions (figure 4). These findings were replicated in vivo by performing intraneural injections. Again, only IgG4 antibodies were found to penetrate the paranodal regions. Of interest, the level of antibody penetration across the paranodal region was similar at 1 or 3 days after intraneural injection, and IgG4 deposition was detected only at the border of the nodes of Ranvier (figure 4). No paranodes presented a complete invasion by IgG4 at 1 or 3 days postinjection.

## Discussion

Antibodies to nodal/paranodal protein were found, albeit with a low frequency, in Italian patients with CIDP and are associated with target- and isotype-specific clinical features, as previously reported. The prevalence of anti-Nfasc155 and anti-CNTN1 antibodies was similar to that reported by previous studies in European patients but was lower compared with that in Japanese patients.^5,10^ The discrepancy may be attributable to differences in inclusion criteria, ethnicities, and nonstandardized laboratory techniques. The frequency of anti-Caspr1 IgG4 antibodies was equal to that of antibodies against CNTN1, confirming that Caspr1 may also represent a relevant target of the immune response in Caucasian patients with CIDP. None of the healthy or pathologic controls tested, including patients affected by other neuropathies, showed reactivity against Nfasc155, CNTN1, or Caspr1. These maximum levels of specificity entail that the tests perform very well for the diagnosis of such CIDP subtypes.

In addition, it is worth noting that the results of antibody testing by ELISA and CBA were concordant in all samples across the 2 testing centers in Pavia and Montpellier, indicating that these techniques, although not broadly available, have a high reproducibility if performed in specialized centers.

Our findings largely confirm previous observations by several groups showing a tight clinical serologic correlation of antibodies to nodal/paranodal proteins and clinical features of seropositive patients with CIDP.

The patients with anti-Nfasc155 IgG4 antibodies showed earlier onset, distal predominant lower limb weakness, and gait disturbance. Tremor was also present, although only in 1 case this was disabling. One patient with such antibodies developed bilateral optic neuritis during steroid tapering 18 months after the onset of the neuropathy.

The patients with anti-CNTN1 antibodies were older and showed a subacute or rapidly progressive severe sensory and motor neuropathy. Previous studies found that anti-CNTN1 antibodies were associated with either predominant motor or sensory impairment. Here, both sensory and motor fibers were equally severely affected, and the patients became wheelchair dependent few months after the disease onset. Notably, 1 patient with anti-CNTN1 antibodies of IgG3 and IgG4 subclasses showed the contemporary occurrence of membranous glomerulonephritis. This association had been reported in 3 cases with anti-CNTN1 antibodies,^7,22,23^ and either a direct damage or indirect damage, after the deposition of immune complexes, can be hypothesized. Of interest, a recent study showed that complement deposition may contribute to the pathophysiology of anti-CNTN1-associated neuropathy, particularly in patients with a predominance of the IgG3 subclass.^24^

Neuropathy in patients with anti-Caspr1 IgG4 antibodies was also highly debilitating. Pain did not seem to be a clinical feature associated with the presence of anti-Caspr1 antibodies in our series, as opposed to the first report of 2 patients with Caspr1-associated inflammatory neuropathy.^8^

As a novel observation of our study, we found that patients with anti-Nfasc155 or Caspr1 antibodies of the IgG1, IgG3, or undetectable IgG isotype did not show clinical features distinct from seronegative patients. Most importantly, patients with anti-Nfasc155, CNTN1, or Caspr1 IgG4 antibodies, but not patients with antibodies to Nfasc155 or Caspr1 of IgG1, IgG3, or undetectable IgG isotype, showed a significantly lower response rate to IVIG compared with seronegative patients.

Analysis of myelinated fibers in the skin biopsy of patients with anti-Nfasc155, CNTN1, or Caspr1 IgG4 antibodies showed morphological changes of the nodes of Ranvier, including elongation of the node and loss of neurofascin and Caspr1 staining at paranodes, which were absent or less prominent in seronegative CIDP cases or in the case with anti-Nfasc155 antibodies of an undetectable isotype. These data add to other recent histopathologic and neurophysiologic observations in patients with antibodies to nodal/paranodal proteins^14,25^ and indicate that isotype determination appears to be crucial to correctly identify such patients and to guide treatment.

The high frequency of anti-Caspr1 antibodies in our series prompted us to further investigate their pathogenic effects. Albeit the experimental setting was limited to the examination of the effects of anti-Caspr1 autoantibodies from a single patient, we found that anti-Caspr1 IgG4, but not anti-Caspr1 IgG1, antibodies affect the interaction between the CNTN1/Caspr1 and Nfasc155 complex using a cell aggregation assay. These findings are in line with the previously reported pathogenic role of anti-CNTN1 IgG4 antibodies^11,12^ and suggest that IgG4 antibodies targeting the CNTN1/Caspr1 complex may have a function-blocking activity and disrupt the paranodal axo-glial contact. In keeping with this experimental evidence, our data also seem to indicate that anti-Caspr1 IgG4 antibodies can penetrate the paranodal regions. It is worth noting that the pathogenic mechanism of IgG4 antibodies in other diseases implicates the disruption of cell adhesion protein complexes too, by inhibiting protein-protein interaction, and that therapies aiming at downregulating the humoral immune response, such as rituximab, showed some efficacy in anti-Nfasc155- and anti-Caspr1 antibody-associated CIDP, as well as in other IgG4-related diseases, likely because of the depletion of the Nfasc155- or Caspr1-reactive B cells.^26^

Despite the limitation entailed by the retrospective data collection, in the present series, immunosuppressive treatment, including rituximab, cyclophosphamide, and methotrexate, seemed effective in IVIg-resistant IgG4 seropositive patients if started early in the disease course. Of note, we did not detect a significant difference in the response to steroids between patients with anti-paranodal IgG4 antibodies and seronegative patients, confirming steroids as an effective therapeutic option in CIDP cases, independent from their serologic status for anti-Nfasc155, CNTN1, or Caspr1 antibodies. Low numbers of patients treated with plasma exchange, and the temporal overlap of plasma exchange cycles with other treatments (case 12), prevented us from drawing firm conclusions on its role in patients with antiparanodal IgG4 antibodies.

Although patients with antibodies against the nodal/paranodal component often, albeit not always, show additional clinical features, CIDP phenotype was typical in all IgG4 and non-IgG4 seropositive patients according to the EFNS/PNS criteria, whereas none of the sera from atypical CIDP cases or control sera showed the presence of these antibodies. Together, this observation challenges the view that the search for antibodies against the nodal/paranodal component may be limited to atypical CIDP cases.

Testing for the presence of antibodies against Nfasc155, CNTN1, and Caspr1, followed by IgG isotype determination in seropositive cases should be part of the diagnostic workup in inflammatory neuropathies to improve diagnostic accuracy and guide treatment. Moreover, knowledge of the mechanism underlying these CIDP subtypes might shed light on the pathophysiology and help further understanding of this complex and heterogeneous disease.

## Glossary

Caspr1: contactin-associated protein 1
CBA: cell-based assay
CIDP: chronic inflammatory demyelinating polyradiculoneuropathy
CNTN1: contactin-1
EFNS/PNS: European Federation of Neurological Societies/Peripheral Nerve Society
GBS: Guillain-Barré syndrome
GFP: green fluorescent protein
HC: healthy control
IVIG: intravenous immunoglobulin
MMN: multifocal motor neuropathy
MRC: medical research council
Nfasc155: neurofascin-155
ONLS: Overall Neuropathy Limitation Scale
PN: peripheral neuropathy
